# Potent Cell-Intrinsic Immune Responses in Dendritic Cells Facilitate HIV-1-Specific T Cell Immunity in HIV-1 Elite Controllers

**DOI:** 10.1371/journal.ppat.1004930

**Published:** 2015-06-11

**Authors:** Enrique Martin-Gayo, Maria Jose Buzon, Zhengyu Ouyang, Taylor Hickman, Jacqueline Cronin, Dina Pimenova, Bruce D. Walker, Mathias Lichterfeld, Xu G. Yu

**Affiliations:** 1 Ragon Institute of MGH, MIT and Harvard, Boston, Massachusetts, United States of America; 2 Infectious Disease Division, Massachusetts General Hospital, Boston, Massachusetts, United States of America; 3 Howard Hughes Medical Institute, Chevy Chase, Maryland, United States of America; 4 Infectious Disease Division, Brigham and Women’s Hospital, Boston, Massachusetts, United States of America; Emory University, UNITED STATES

## Abstract

The majority of HIV-1 elite controllers (EC) restrict HIV-1 replication through highly functional HIV-1-specific T cell responses, but mechanisms supporting the evolution of effective HIV-1-specific T cell immunity in these patients remain undefined. Cytosolic immune recognition of HIV-1 in conventional dendritic cells (cDC) can facilitate priming and expansion of HIV-1-specific T cells; however, HIV-1 seems to be able to avoid intracellular immune recognition in cDCs in most infected individuals. Here, we show that exposure of cDCs from EC to HIV-1 leads to a rapid and sustained production of type I interferons and upregulation of several interferon-stimulated effector genes. Emergence of these cell-intrinsic immune responses was associated with a reduced induction of SAMHD1 and LEDGF/p75, and an accumulation of viral reverse transcripts, but inhibited by pharmacological blockade of viral reverse transcription or siRNA-mediated silencing of the cytosolic DNA sensor cGAS. Importantly, improved cell-intrinsic immune recognition of HIV-1 in cDCs from elite controllers translated into stronger abilities to stimulate and expand HIV-1-specific CD8 T cell responses. These data suggest an important role of cell-intrinsic type I interferon secretion in dendritic cells for the induction of effective HIV-1-specific CD8 T cells, and may be helpful for eliciting functional T cell immunity against HIV-1 for preventative or therapeutic clinical purposes.

## Author Summary

Elite controllers (EC), a small group of HIV-1-infected individuals that are able to control viral replication in the absence of antiretroviral therapy, provide living evidence that the human immune system is able to spontaneously control HIV-1 infection and serve as a model for inducing a functional cure of HIV-1 infection in broader patient populations. Prior studies indicated that T cell-mediated immune responses represent the backbone of effective antiviral immune defense in EC; however, emerging studies suggest that innate and cell-intrinsic immune activities may have critical roles for supporting and enhancing HIV-1-specific T cells. Here, we performed a detailed investigation of conventional dendritic cells (cDC) from elite controllers and their responses to HIV-1 infection. These studies indicate that cDC from EC have improved abilities to sense cytosolic HIV-1 replication products, and can more effectively mount cell-intrinsic type I interferon (IFN) secretion in response to HIV-1 infection. Notably, such increased production of type I IFN in response to viral antigen translated into enhanced abilities to prime and expand HIV-1-specific T cells. Together, these data suggest that a fine-tuned interplay between innate dendritic cell responses and adaptive HIV-1-specific CD8 T cells represents a critical component of antiviral immune defense in elite controllers.

## Introduction

Elite controllers can maintain undetectable levels of HIV-1 viral replication in the absence of antiretroviral therapy, at least in part through the generation of highly-efficient HIV-1-specific T cell responses [1–4]. As such, these patients provide living evidence that in principle, the human immune system is capable of generating a T cell-mediated immune response that allows to effectively controlling HIV-1 replication. However, it is uncertain why such immune responses occur only in very few patients, and what mechanisms support the development of highly-effective T cell immune responses in such a small number of individuals, and not in the majority of alternative individuals. Dendritic cells (DCs) represent the most effective naturally-occurring antigen-presenting cells and have critical roles for inducing and maintaining antigen-specific T cell responses [5–10]. However, specific functional characteristics of DCs that are instrumental in generating protective HIV-1-specific T- cell responses in elite controllers are unclear, and represent an understudied area of investigation. Understanding the mechanisms that facilitate the induction of effective HIV-1-specific T cells by dendritic cells is of critical interest for developing improved immunologic approaches for HIV-1 treatment and prevention, specifically since most HIV-1 vaccine candidates rely on dendritic cells for inducing HIV-1-specific immune responses.

Human cells have the ability to respond to viral infections by cell-intrinsic immune responses that lead to secretion of type I interferons (IFN-I) and upregulation of a wide panel of IFN-stimulated genes (ISG) with antiviral effector functions [11,12]. This cell-intrinsic immune response is extremely effective in defending the host against a panel of different viruses [13], but early reports suggested that human cells are unable to mount such immune responses against HIV-1 [14,15]. However, recent discoveries have shown that human conventional DCs (cDC) are generally capable of generating IFN-I responses to HIV-1, but HIV-1 seems to be able to escape from such cell-intrinsic immunity in most patients [16–18]. A dominant mechanism that may allow HIV-1 to avoid cell-intrinsic immune responses in cDC includes the expression of SAMHD1, a host protein that can block HIV-1 reverse transcription by hydrolyzing dNTPs [19–22] or inhibit HIV-1 RNA through direct degradation [23]. In the presence of experimental SAMHD1 knockdown, viral replication in host cells progresses beyond the level of reverse transcription, and viral reverse transcripts or proteins can be sensed by host molecules that can initiate secretion of type I interferons [16,24–27]. In this way, restriction of HIV-1 replication by SAMHD1 may paradoxically benefit the virus more than the host, which likely explains why SAMHD1 represents the only effective HIV-1 restriction factor that HIV-1 does not neutralize through the activity of accessory proteins [28]. In addition to SAMHD1, cell-intrinsic immune responses in DCs are inhibited by the host protein TREX1, a host exonuclease that degrades HIV-1 reverse transcripts, which otherwise trigger microbial DNA sensors, leading to cell-intrinsic secretion of type I interferons [18,29].

In the present study, we demonstrate that unlike chronic progressors, dendritic cells from elite controllers have the ability to effectively mount cell-intrinsic type I IFN secretion in response to HIV-1 infection, likely through an accumulation of viral reverse transcripts that serve as substrates for the cytosolic DNA sensor cGAS (cyclic guanosine monophosphate-adenosine monophosphate (GMP-AMP) synthase). Recognition of these early viral replication products in elite controllers seemed to be facilitated by an enhanced upregulation of cGAS, led to rapid and sustained secretion of type I IFNs, and was functionally relevant for supporting effective HIV-1-specific CD8 T cell responses. Together, these results suggest previously unrecognized innate mechanisms of HIV-1 immune recognition that contribute to T cell-mediated immune control of HIV-1.

## Results

### Exposure to HIV-1 induces rapid maturation and sustained type I IFN secretion in conventional DCs from elite controllers

**Fig 1:**
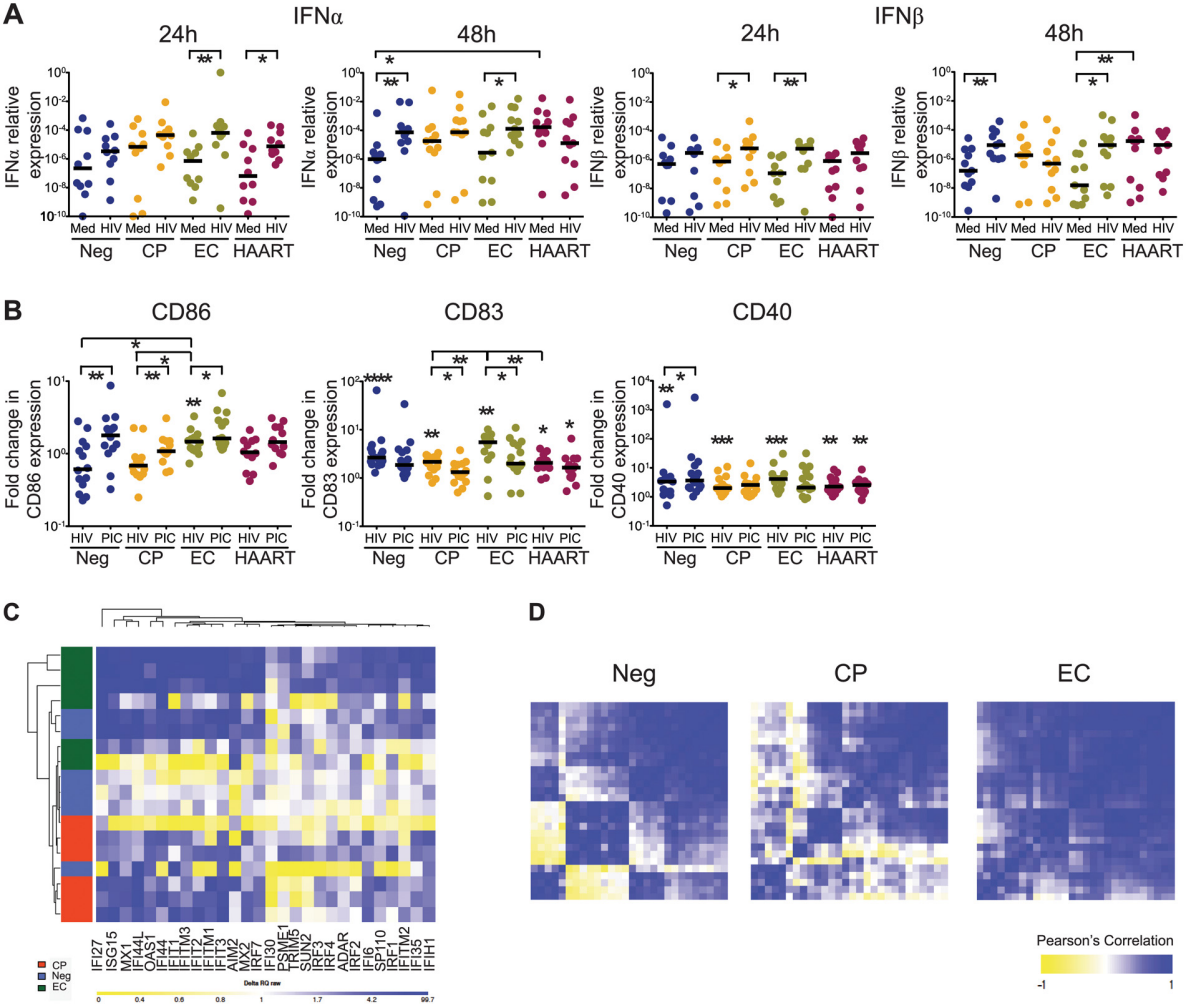
Type I IFN secretion and activation in cDCs after *ex-vivo* exposure to HIV-1. (A): IFNα and IFNβ mRNA levels in isolated BDCA1^+^ cDCs from HIV-negative persons (Neg), individuals with chronic progressive HIV-1 infection (CP), Elite controllers (EC) and HAART-treated HIV-1 patients (HAART) at 24 and 48 hours after exposure to HIV-1 (HIV) or to media only (Med) as negative control. Horizontal lines represent the median for each specific cohort and experimental condition. (B): Mean Fluorescence Intensity (MFI) reflecting surface expression of CD86, CD83 and CD40 in cDCs from the different study cohorts at 24 hours after infection with HIV-1 (HIV) or after exposure to poly(I:C) (PIC). MFI values are expressed as fold-changes in comparison to baseline levels. Intra-individual differences were tested for statistical significance using Wilcoxon matched-pairs signed rank tests (above each cohort), differences between cohorts were tested using a Kruskal-Wallis test with post-hoc Dunn’s test; * p<0.05; ** p<0.01; *** p < 0.001; **** p< 0.0001. Horizontal lines represent the median for each specific cohort and experimental condition. (C): Heatmaps reflecting gene expression patterns of 28 interferon-stimulated genes (ISG) in cDCs from Neg (n = 6), CP (n = 6) and EC (n = 6) at 48 hours after infection with HIV-1. (D): Heatmaps reflecting correlations among gene expression intensities of all 28 ISGs in indicated study cohorts. Color-coding reflects Pearson’s correlation coefficient indicating strengths of statistical association between expression intensities of given gene pairs.

To determine whether human primary cDCs from EC are capable of mounting cell-intrinsic type I IFN responses against HIV-1, we conducted *ex-vivo* infection experiments with PBMC from different study cohorts with a GFP-encoding VSV-G pseudotyped HIV-1 virus causing single rounds of viral infections [16]; this viral construct infects cells independently of viral coreceptor-mediated entry processes, but can be intracellularly sensed in a similar way as R5-tropic primary HIV-1 isolates [27]. At 24 and 48 hours after infection, cDCs were isolated from PBMC, and subjected to gene expression analysis. Cells from untreated, chronically HIV-1-infected patients (CP), HIV-1-infected patients receiving suppressive antiretroviral therapy (HAART) and HIV-1-uninfected persons were used for comparison. These experiments demonstrated that upon exposure to HIV-1, IFNα and IFNβ mRNA expression was rapidly and significantly upregulated at 24 hours post-infection (p.i.) in cDCs from EC; these elevated levels were sustained at 48h p. i. (Fig 1A). Exposure to control viral preparations (generated by transfection of the viral producer cell line with salmon sperm DNA instead of the HIV-1 plasmid) failed to induce type I IFN responses. In contrast to cDC from EC, upregulation of IFNα/β expression in cDC from HIV-1 negative persons was delayed and was not detectable at statistically significant levels until 48h p.i.. IFNα expression in cDCs from HAART-treated patients after HIV-1 exposure was only transiently induced at 24 hours p.i. (Fig 1A); IFNβ expression was barely affected by HIV-1 infection in this cohort. In untreated CP, baseline levels of IFNα/β in cDCs were slightly elevated in comparison to alternative patients, but did not increase substantially upon HIV-1 exposure, neither at 24 hours nor 48 hours p. i.

We subsequently analyzed the surface expression of costimulatory molecules and activation markers on cDCs after HIV-1 infection. We observed that the rapid and sustained upregulation of type I IFN expression in cDCs from EC after HIV-1 infection was associated with a significantly increased surface expression of CD86, CD83 and CD40 at 24 hours p.i., which reached levels otherwise observed after stimulation of cDC with the TLR3 ligand Poly(I:C) (Fig 1B and S1 Fig). cDCs from all the alternative study cohorts also upregulated costimulatory molecules and maturation markers at 48 hours after infection, indicating that they were capable of responding to the virus, but induction of these molecules was delayed in comparison to cDC from EC (S1B Fig). Addition of exogenous IFNβ to the media culture induced the expression of costimulatory molecules in a dose-dependent manner (S1C Fig) and rescued the cDC maturation defect observed in CP and HAART patients (S1D Fig). In addition, we observed that the expression intensity of 28 interferon-stimulated effector genes (ISG) was upregulated in cDC at 48 hours post-infection; this occurred in all patient cohorts, but was most strongly and consistently noticeable in cDC from EC, despite some heterogeneity within this group (S1 and S2 Tables and Fig 1C) [30]. Induction of most ISGs in cDCs from ECs occurred in a highly interconnected and coordinated fashion, while expression of individual ISG in CP and HIV-1 negative persons were more weakly correlated, and exhibited opposing increases and decreases for certain groups of transcripts (Fig 1D). Together, these findings demonstrate a unique kinetic profile of activation, maturation and IFN secretion in cDCs from EC after exposure to HIV-1, and suggest that cDCs from EC differ from those of other patients by their ability to mount rapid and sustained cell-intrinsic secretion of IFNα/β in response to HIV-1.

### Accumulation of HIV-1 RT products in cDCs from EC

**Fig 2:**
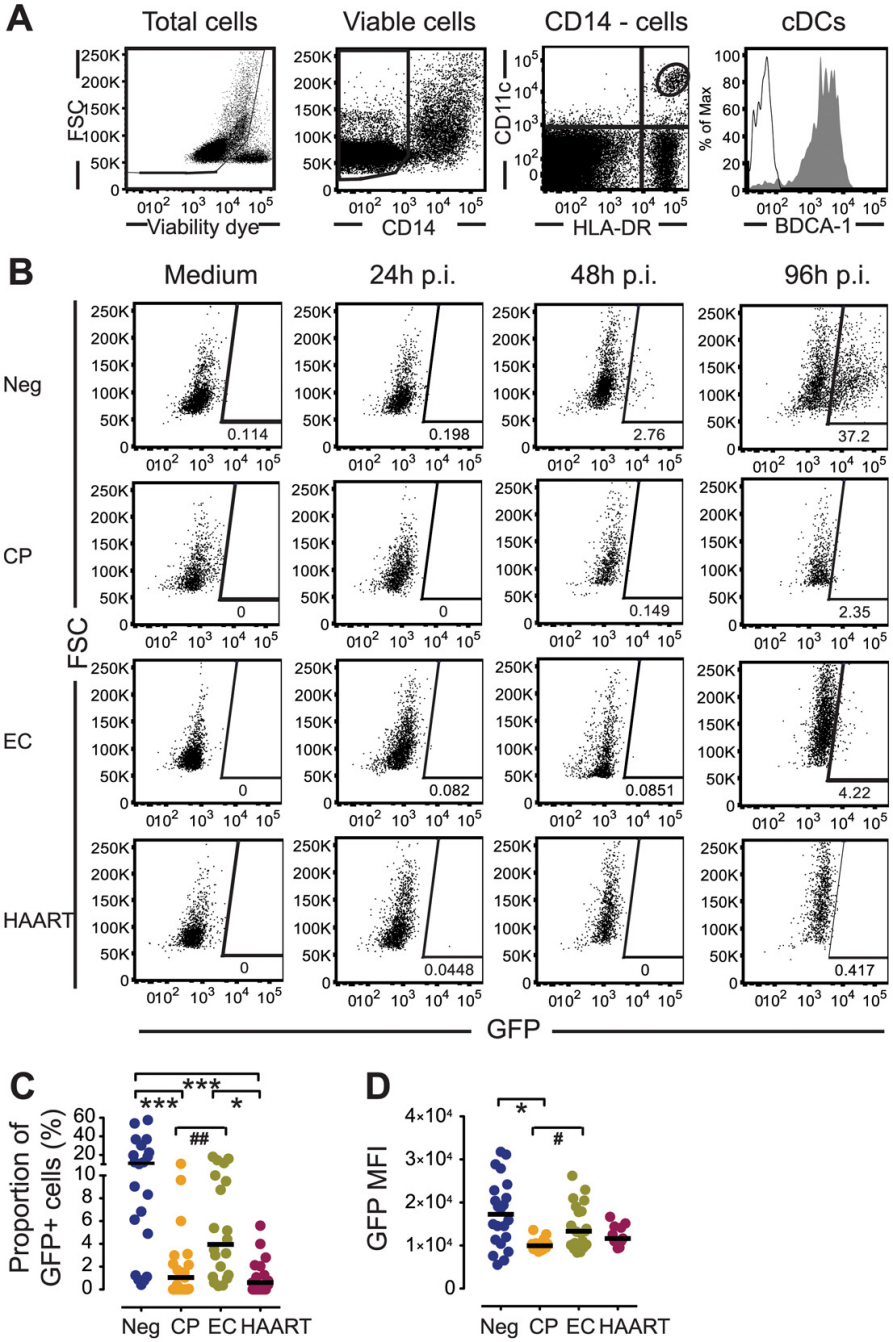
Susceptibility of primary cDCs to *ex-vivo* infection with HIV-1. (A): Flow cytometry gating strategy for defining cDCs in bulk PBMC cultures. (B): Representative flow cytometry dot plots indicating GFP expression in gated CD11c^+^ HLA-DR^+^ cDCs from Neg, CP, EC and HAART individuals after 24, 48 and 96 hours of *ex-vivo* infection with GFP-encoding HIV-1. Numbers in dot plots reflect the proportion of GFP-positive cells within gated cDCs. (C–D): Proportion (C) and GFP MFI (D) of GFP^+^ cDCs from Neg, CP, EC and HAART subjects at 96 hours after infection with HIV-1 (n = 20 tested subjects for each cohort). Horizontal lines represent the median for each specific cohort and experimental condition. Differences among cohorts were tested using a Kruskal-Wallis test with post-hoc Dunn’s test (* p<0.05; *** p<0.001) or using Mann Whitney U test (# p<0.05; ## p<0.01).

To explore the underlying reasons for the ability of cDCs from EC to mount early and strong cell-intrinsic immune responses upon HIV-1 infection, we subsequently analyzed HIV-1 replication steps in cDCs from the different patient cohorts. In agreement with previous reports [16], productively infected cells were hardly detected in primary cDCs during the first 48 hours after exposure to HIV-1 (Fig 2A and 2B), irrespectively of the patient cohorts. Such resistance to HIV-1 was also observed in monocyte-derived DC from HIV-1 negative persons (S2A Fig), as reported previously [16]. However, we noted significant proportions of GFP-expressing, HIV-1 positive cells in primary cDCs from HIV-negative persons at 96 hours p.i. (Fig 2B and 2C) when MDDC continued to resist productive HIV-1 infection (S2A Fig). Thus, consistent with previous studies [31–33], our data indicate that primary cDCs from HIV-1 negative subjects are capable of supporting HIV-1 replication, at least after prolonged periods of incubation. Interestingly, cDCs from both CP and HAART-treated patients displayed substantially reduced susceptibility to productive infection (Fig 2B and 2C). Remarkably, cDCs from EC also seemed less susceptible to HIV-1 infection than cDCs from healthy individuals, but a trend towards higher proportions of infected cells (Fig 2C) and higher per-cell levels of GFP expression (Fig 2D) were found in cells from these patients compared to CP and HAART individuals. Similar results were obtained using a CCR5-tropic GFP-encoding HIV-1 virus, although as expected, efficiency of infection was lower than with the VSV-G-pseudotyped HIV-1 virus (S2B and S2C Fig). Importantly, differential susceptibility of cDCs from each cohort to infection with HIV-1 was not associated with significant differences in cell viability among the study groups (S2D Fig). Together, our findings indicate that primary cDCs can be productively infected with HIV-1 *in vitro* and paradoxically suggest that cDCs from EC subjects are more susceptible to *de novo* infection with HIV-1 than CP and HAART patients.

**Fig 3:**
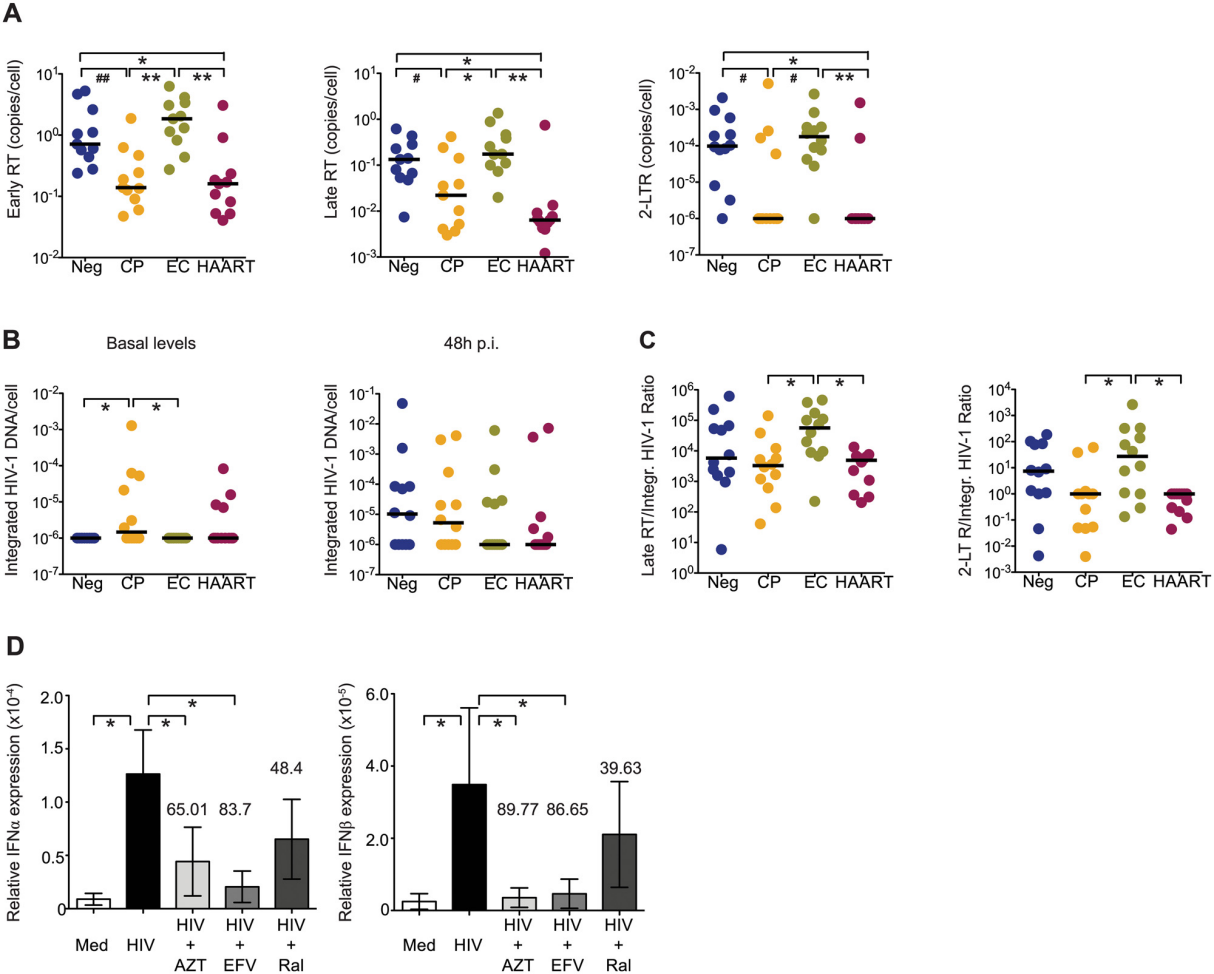
HIV-1 replication patterns in cDCs from EC. (A): Early and late HIV-1 reverse transcripts (RT) and 2-LTR circles in cDCs from indicated study subjects at 48 hours after *ex vivo* infection with HIV-1. (B): Analysis of integrated HIV-1 DNA in primary cDCs from the different study cohorts. Basal levels of integrated HIV-1 DNA are shown in the left panel, right panel shows *de novo* HIV-1 integration in cDCs after subtraction of baseline levels of integrated HIV-1 DNA. (C): Analysis of late HIV-1 RT products and 2-LTR circles normalized to levels of *de novo* integrated HIV-1 DNA in cDCs at 48 hours after infection. (A–C): Differences between different cohorts were tested for statistical significance using a Kruskal-Wallis test with post-hoc Dunn’s test or using Mann Whitney U test (# p<0.05; ## p<0.01). (A,B,C). Horizontal lines represent the median for each specific cohort and experimental condition. (D): Inhibition of IFNα and IFNβ mRNA expression in cDCs from EC cultured in media (Med) or infected with HIV-1 (HIV) in the presence or absence of AZT, Efavirenz (EFV) or Raltegavir (Ral). Data reflect mean and standard error from qPCR values of IFNα and IFNβ mRNA levels after normalization to β-actin endogenous expression from n = 5 experiments. Numbers above bars represent the mean percentage of inhibition induced by each drug. Differences were tested for statistical significance using a one-tailed Wilcoxon matched-pairs signed rank test, * p<0.05.

We next analyzed early steps of viral replication in primary cDCs from our study cohorts at 48 hours p.i.. Notably, despite significantly lower levels of productive HIV-1 infection in cDCs from EC compared to HIV-1 negative individuals, cDCs from EC contained similar levels of both early and late reverse transcripts and 2-LTR circles as those from HIV-1 negative individuals; however, in comparison to cDCs from CP and HAART-treated patients, RT products and 2-LTR circles were significantly elevated in cDC from EC (Fig 3A). Importantly, such differences in HIV-1 RT product concentrations among the different study cohorts were already evident at 24h p.i. (S3 Fig). In contrast, no significant difference was found in levels of integrated HIV-1 DNA in cDCs from EC in comparison to other study cohorts (Fig 3B). These data resulted in significantly increased ratios of late RT transcripts and 2-LTR DNA to integrated HIV-1 DNA in EC compared to CP and HAART-treated cohorts, consistent with a disproportionate accumulation of RT products in cDCs from EC relative to other study cohorts (Fig 3C). Therefore, our data suggest that elevated levels of viral RT products present in cDCs from EC might facilitate cell-intrinsic viral recognition and lead to enhanced production of type I IFNs in response to HIV-1. To test whether type I IFN responses were in fact dependent on the presence of HIV-1 RT products, we infected cDCs from EC with HIV-1 in the presence or absence of antiretroviral drugs inhibiting either early or late steps of HIV-1 reverse transcription or viral DNA integration. As shown in Fig 3D, pharmacological inhibition of HIV-1 reverse transcription dramatically reduced the expression of type I IFNs in primary cDCs. In contrast, inhibition of HIV-1 integration had only a modest effect on the expression of type I IFNs that did not reach statistical significance. Overall, these findings indicate distinct viral replication patterns in cDCs from EC that lead to a relative accumulation of RT products in cDCs, and are associated with a more effective induction of cell-intrinsic type I IFN responses.

### Weak induction of SAMHD1 and LEDGF/p75 in cDCs from EC

**Fig 4:**
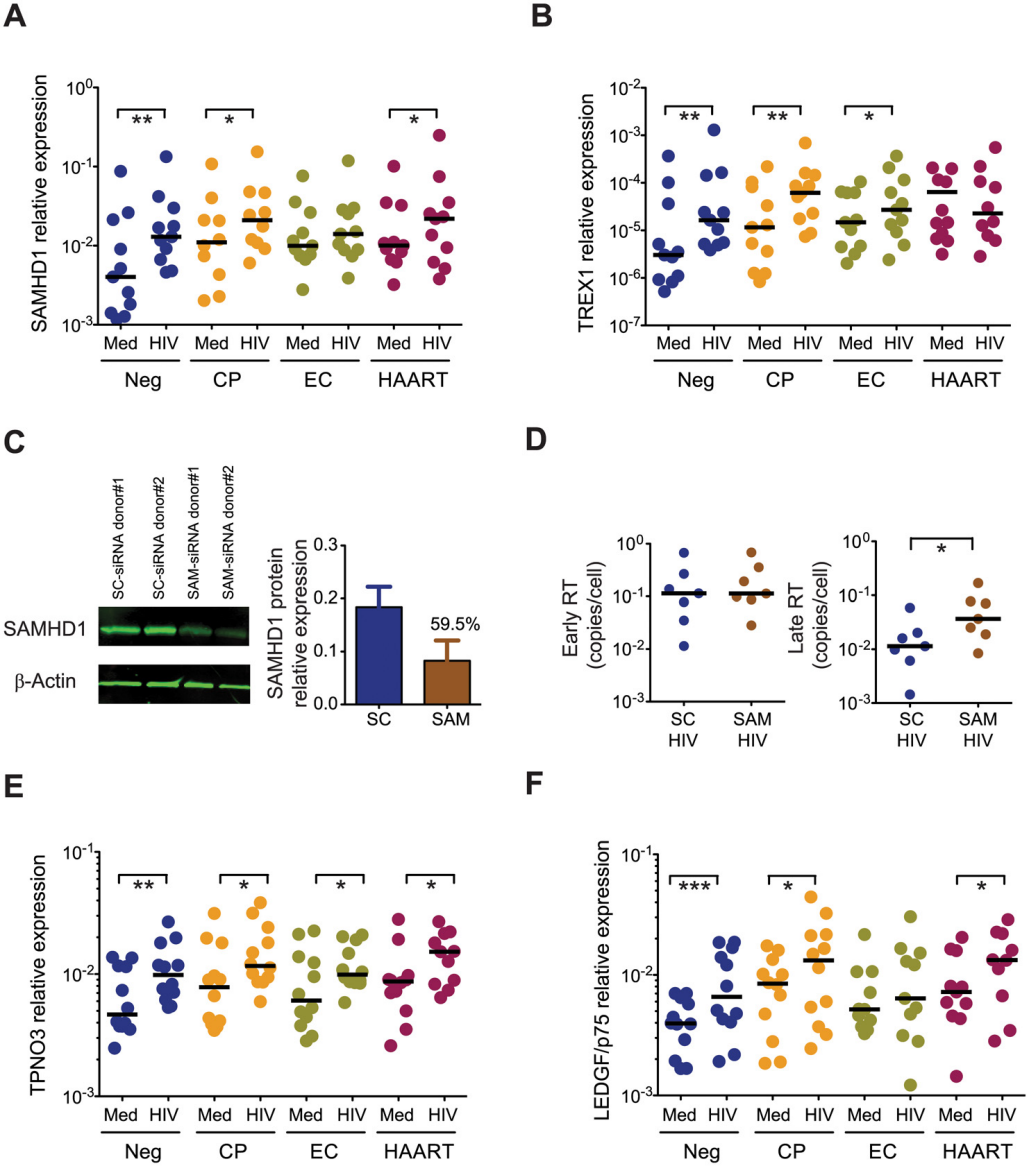
Expression of host restriction factors in cDCs from EC. (A) SAMHD1 mRNA expression levels in cDCs from Neg (n = 11), CP (n = 11), EC (n = 11) and HAART-treated (n = 11) subjects at 48 hours after exposure to HIV-1 (HIV) or to media only (Med). (B): mRNA levels of TREX1 in cDCs from Neg (n = 11), CP (n = 11), EC (n = 11) and HAART (n = 11) individuals at 48 hours after exposure to HIV-1 (HIV), or to media only (Med). (A–B): Horizontal lines represent the median for each specific cohort and experimental condition. (C): Efficiency of siRNA-mediated SAMHD1 protein knock-down in primary cDCs 24h after treatment with scramble (SC) or SAMHD1-specific (SAM) siRNAs. Left panel shows a representative western blot analysis of SAMHD1 and β-actin expression on cDCs, right panel represents cumulative SAMHD1 expression data after normalization to β -actin in DCs nucleofected with SC (blue; n = 4) or SAM (brown; n = 4) siRNAs. The number above the bar represents the mean inhibition (%) in SAMHD1 protein expression after treatment with specific siRNAs. (D): Relative expression of early (left panel) and late (right panel) HIV-1 reverse transcripts in cDCs after *ex vivo* infection in the presence or absence of siRNA-mediated SAMHD1 silencing. Statistical significance was calculated using a Wilcoxon matched-pairs signed rank test. (E-F): mRNA levels of TPNO3 (E) and LEDGF/p75 (F) in cDCs from the indicated study cohorts at 48 hours after exposure to HIV-1 (HIV), or to media only (Med). Differences within cohorts were calculated using a Wilcoxon matched-pairs signed rank test * p<0.05; ** p<0.01; *** p<0.001. (D-E-F): Horizontal lines represent the median for each specific cohort and experimental condition.

We hypothesized that altered expression patterns of intracellular host proteins might be responsible for the accumulation of HIV-1 RT products observed in cDCs from EC. To investigate this, we first analyzed the expression of SAMHD1 and TREX1 in cDCs after exposure to HIV-1; these host factors can affect HIV-1 RT products by reducing the synthesis of HIV-1 RT products [19–22] or enhancing their degradation [18,29], respectively. Basal mRNA expression levels of SAMHD1 and TREX1 were not significantly different in cDCs from the different patient cohorts, although a tendency for higher expression was detected in cDCs from HIV-1-infected patients (Fig 4A and 4B). However, after 48 hours of exposure to HIV-1, only cDCs from HIV-1 negative study persons, HAART and CP significantly induced the transcription of SAMHD1, while cDC from ECs displayed largely similar mRNA levels of SAMHD1 as uninfected cDCs (Fig 4A). Importantly, similar patterns of SAMHD1 protein expression were also observed when cDCs were isolated prior to exposure to HIV-1 (S4A and S4B Fig). Notably, siRNA-mediated downregulation of SAMHD1 in *ex-vivo* infected cDCs led to an accumulation of late RT products (Fig 4C and 4D). With the exception of HAART-treated patients, expression levels of TREX1 were significantly increased in cDCs from all other study cohorts after HIV-1 exposure, although this was less obvious in cDCs from EC (Fig 4B). Together, these data suggest that a weaker induction of SAMHD1, and possibly of TREX1, might facilitate HIV-1 replication and contribute to accumulation of HIV-1 RT products in cDCs from EC after *ex vivo* infection.

Since the efficiency of HIV-1 DNA integration may also affect the described accumulation of HIV-1 RT products in cDCs from EC, we subsequently analyzed transcriptional levels of host proteins supporting HIV-1 integration, such as [34–36] TPNO3 [37,38] and LEDGF/p75 [39,40]. As shown in Fig 4E, a significant increase in the amounts of TPNO3 transcripts was observed in cDCs from all patient cohorts after exposure to HIV-1. In contrast, LEDGF/p75 mRNA expression remained essentially unchanged in cDCs from EC, as opposed to cDCs from the other three patient cohorts in which upregulation of LEDGF/p75 occurred; this suggests that ineffective LEDGF/p75-dependent HIV-1 DNA integration in cDCs from EC may also contribute to an accumulation of RT transcripts that cannot effectively integrate into chromosomal DNA (Fig 4F). Thus, these data indicate that cDCs from EC have unique expression profiles of host factors relevant for shaping and structuring the HIV-1 replication cycle, and suggest that a weak induction of the host restriction factor SAMHD1, and possibly TREX1 and LEDGF/p75, in cDCs from EC may play an important role for a relative accumulation of viral reverse transcripts in cDCs from such patients.

### Cell-intrinsic type I IFN secretion in primary cDCs depends on cGAS

**Fig 5:**
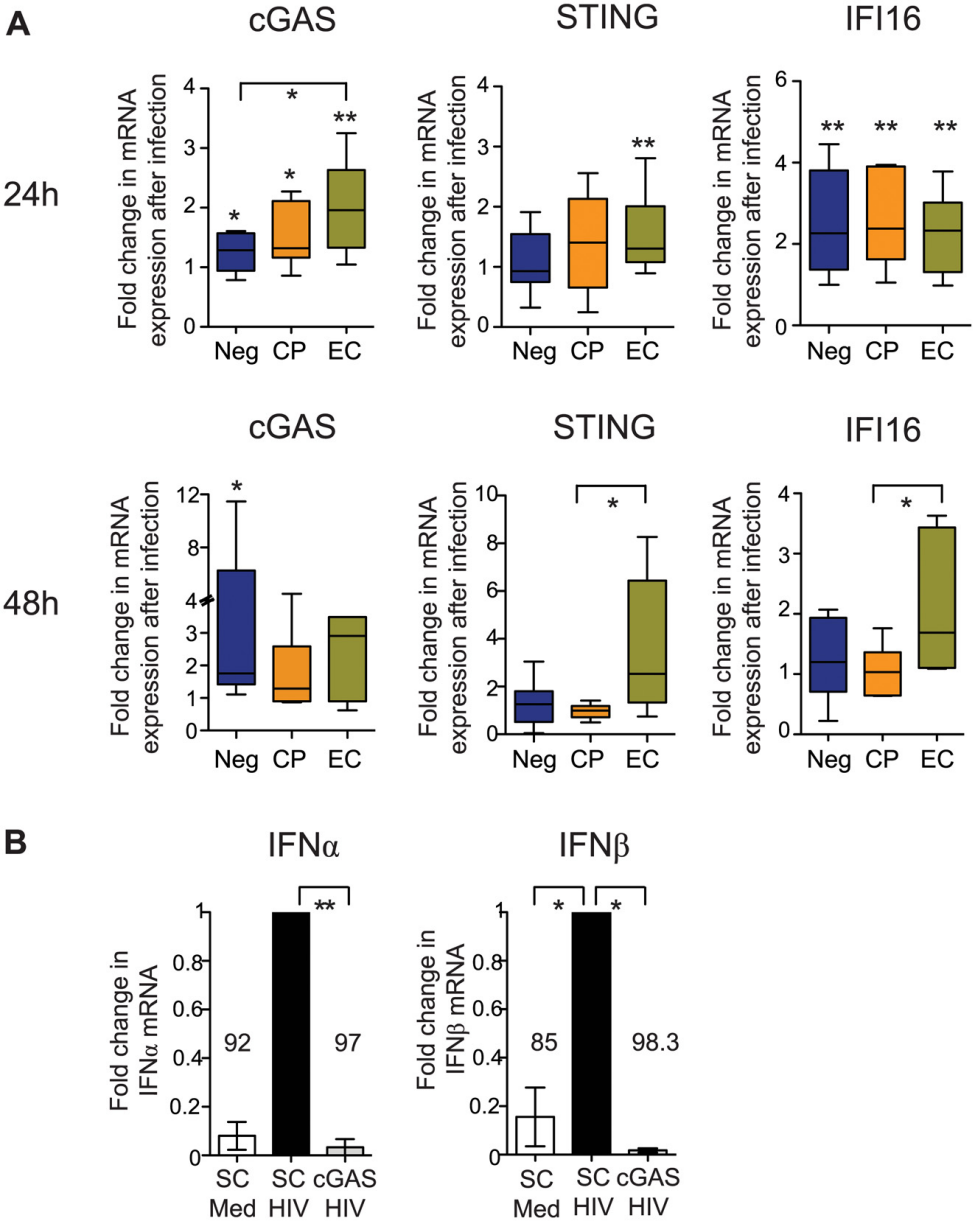
Induction of cytosolic DNA sensors in cDCs from EC. (A): Fold change in cGAS, STING and IFI16 mRNA expression levels in indicated study cohorts at 24 (upper panels) and 48 (lower panels) hours after *ex-vivo* infection with HIV-1. Induction of mRNA expression in comparison to baseline levels was tested for statistical significance using Wilcoxon matched-pairs signed-rank test tests. Significant differences between distinct cohorts were calculated using a Mann Whitney test. No correction for multiple comparisons was applied (B): IFNα and IFNβ expression in primary cDCs nucleofected with scrambled (SC) or cGAS-specific siRNAs, followed by infection with HIV-1. Data from n = 4 experiments are shown. Data were normalized to results from experiments with scrambled siRNA sequences. Differences in type I IFN responses between untreated or SC- or cGAS-nucleofected cDCs were tested for statistical significance using a Kruskal-Wallis test with post-hoc Dunn’s test * p<0.05; ** p<0.01.

We next investigated specific sensors of microbial DNA that may be involved in enhanced cell-intrinsic type I IFN secretion in response to HIV-1 infection in primary cDCs from EC. For this purpose, expression of cytosolic DNA sensors was analyzed at 24 and 48 hours after infection of cDCs from different patient cohorts with HIV-1. Interestingly, we observed that the expression of cGAS, a recognized sensor for HIV-1 DNA in monocyte-derived DC [27,41,42], was more efficiently upregulated in cDCs from EC shortly after infection, but expression levels in alternative patients approached those of EC after 48 hours p.i. (Fig 5A). Interestingly, the expression of STING, a downstream effector of the DNA sensor cGAS [43], was also significantly induced in cDCs from EC at 24 hours p.i. (Fig 5A). Expression of IFI16, a DNA sensor important for the cell-intrinsic recognition of HIV-1 DNA in CD4 T cells [44–46], was similarly induced in cDCs from all cohorts at 24 hours p. i. (Fig 5A). Interestingly, expression of STING continued to increase at 48 hours in EC, but remained largely stable in the other study patient cohorts (Fig 5A). Since STING expression is sensitive to type I interferons, its continuous induction in cDCs from EC may result from stronger cell-intrinsic secretion of IFNα/β described above. Importantly, induction of STING and cGAS appeared to be positively correlated with upregulation of IFNβ at 48 hours p.i. on cDCs (S5A Fig), suggesting an important role of these molecules in cytoplasmic HIV-1 immune recognition. To better define the role of cGAS for the induction of type I IFN responses in cDCs from EC, we analyzed changes of type I IFN expression in HIV-1-infected primary cDCs after siRNA-mediated downregulation of cGAS expression. As shown in Fig 5B, silencing of cGAS (S5B and S5C Fig) drastically impaired the induction of both IFNα and IFNβ secretion in response to HIV-1 infection. These effects were not associated with changes in cell viability (S5B Fig) or an inability to respond to other stimuli such as Poly I:C (S5D Fig) in cDCs. Overall, these data suggest that the HIV-1 DNA sensor cGAS is preferentially upregulated in cDCs from EC, and facilitates a more rapid and efficient recognition of cytoplasmic HIV-1 DNA, leading to more potent cell-intrinsic IFN secretion in these cells.

### Cell-intrinsic immune responses in cDCs from EC support HIV-1-specific T cells

**Fig 6:**
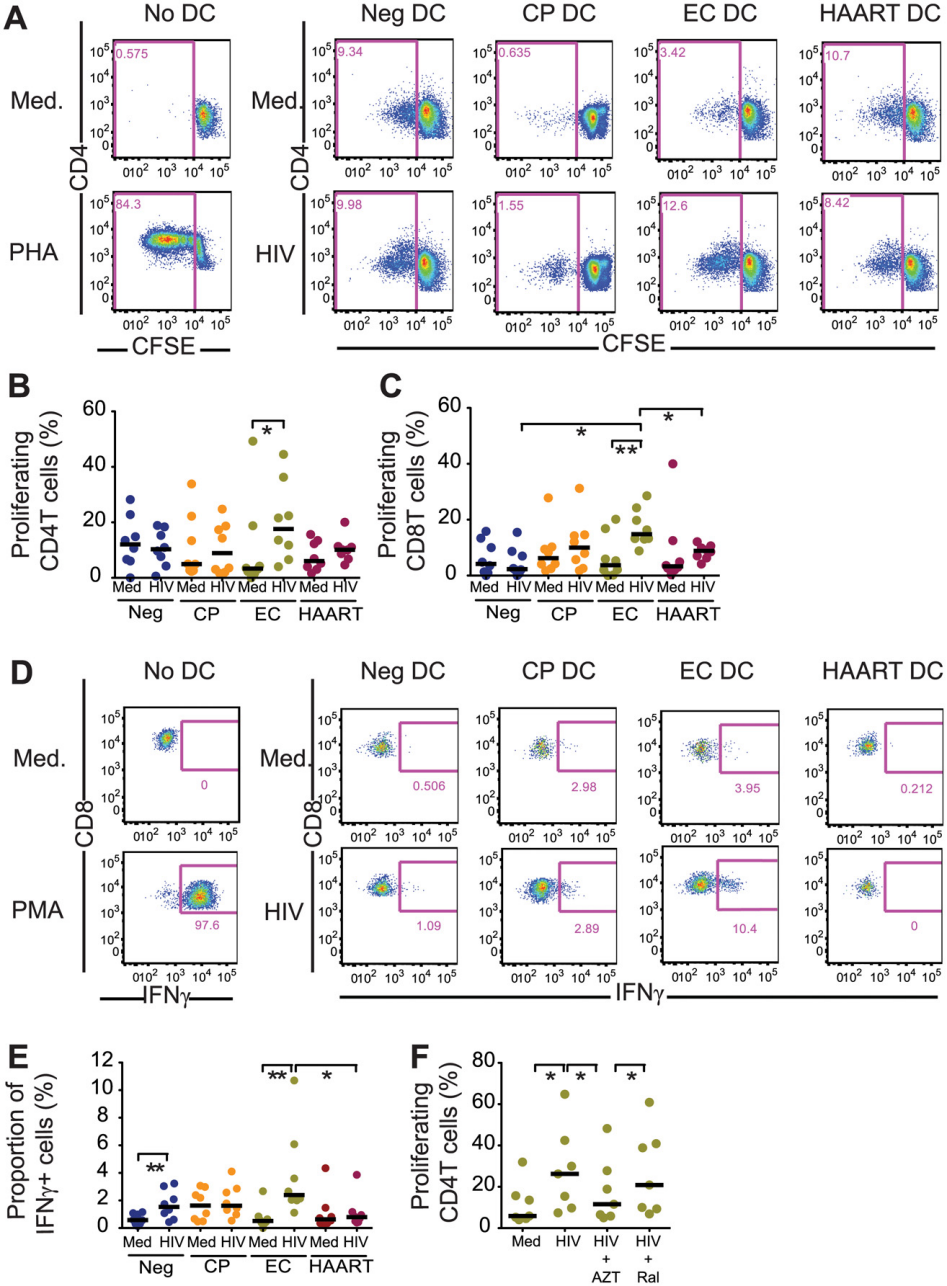
Antigen-presenting properties of cDCs after exposure to HIV-1. (A): Representative flow cytometry plots reflecting proliferation of CD4^+^ T cells after stimulation with allogeneic uninfected (Med) or HIV-1 infected (HIV) cDCs from Neg, CP, EC and HAART patients. Numbers in flow cytometry plots represent the proportion of proliferating CFSE^low^ T cells. (B–C): Induction of allogeneic CD4 (B; n = 8) and CD8 T (C; n = 7) cell proliferation after exposure to HIV-1 infected cDC from indicated study cohorts. Horizontal lines represent the median for each specific cohort and experimental condition. (D): Representative flow cytometry dot plots reflecting IFN-γ secretion in an HLA-A2-SL9 (SLYNTVATL)-specific CTL cell line after 16 hours of co-culture with uninfected or HIV-1-infected cDCs from Neg, CP, EC and HAART individuals. (E): Proportion of IFNγ^+^ cells in the SL9 CTL cell line after exposure to HIV-1-infected cDCs from indicated study groups. Cumulative data from n = 8 experiments are shown. (B,C,E): Differences within and among study groups were tested for statistical significance using a Wilcoxon matched-pairs signed-rank test rank test or a Mann Whitney test corrected for multiple comparisons using Bonferroni method, respectively. * p<0.05; ** p<0.01. (F): Induction of allogeneic CD4 (left; n = 7) and CD8 (right; n = 7) T cell proliferation after exposure to cDC from EC cultured in the presence of media (Med) or infected with HIV-1 in the presence or absence of AZT or RAL. Statistical significance of differences was tested using a Wilcoxon matched-pairs signed rank test. Bonferroni correction was applied for multiple comparisons.* p<0.05. (E-F): Horizontal lines represent the median for each specific cohort and experimental condition.

We sought to determine whether more efficient cell-intrinsic immune responses against HIV-1 in cDCs from EC can result in improved abilities to stimulate antigen-specific T cells. For this purpose, we first tested the ability of uninfected and HIV-1-infected cDCs from the different study cohorts to induce proliferation of CFSE-labeled allogeneic CD4^+^ and CD8^+^ T cells. As shown in Fig 6A, 6B and 6C, HIV-1-infected cDCs from EC had significantly elevated abilities to induce proliferation of allogeneic CD4^+^ and CD8^+^ T cells after 6 days in culture, compared to HIV-1-infected cDCs from alternative study subjects. To confirm these findings with CD8^+^ T cells specific for HIV-1, we cultured a cytotoxic T cell (CTL) clone that recognizes the immunodominant HIV-1 Gag peptide SLYNTVATL restricted by HLA*02:01 [47] in the presence of unstimulated or HIV-1-infected cDCs from HLA-matched study subjects. As shown in Fig 6D and 6E, HIV-1-infected cDCs from EC were able to elicit significantly more IFNγ secretion in HIV-1-specific CD8^+^ T cells, in contrast to cDCs from chronic progressors or HAART-treated individuals which promoted significantly less efficient IFNγ secretion. Therefore, our results indicate that upon HIV-1 infection, cDCs from EC acquire improved antigen-presenting properties and have increased abilities to stimulate HIV-1-specific T cells. Importantly, improved antigen-presenting properties of cDCs from EC after infection with HIV-1 were abrogated by pharmacological inhibition of HIV-1 reverse transcription, but not by inhibition of HIV-1 integration, consistent with our previous identification of viral reserve transcripts as the main viral substrate required for inducing cell-intrinsic immune responses (Fig 6F). Together, these data suggest an important contribution of innate, cell-intrinsic immune recognition of HIV-1 in cDCs to the generation of potent antiviral T cell immune responses in elite controllers.

## Discussion

Cell-intrinsic secretion of type I IFN represents an efficient antimicrobial immune defense strategy, but the clinical significance of these types of immune responses against HIV-1 remains uncertain. Recent studies show that in principle, human cells have the ability to respond to HIV-1 infection with increased secretion of type I interferons; however, several negative regulators of IFN secretion seem to actively prevent these immune responses in most patients [48–50]. In this study, we have conducted a detailed analysis of cell-intrinsic immune responses to HIV-1 in primary dendritic cells from elite controllers, a small subgroup of patients who control HIV-1 replication in the absence of treatment and arguably represent the best patients for detecting effective immune defense mechanisms against HIV-1 disease progression. Our data indicate that cDCs from EC differed from those of other patients by cell-intrinsic type I IFN secretion that occurred rapidly and sustainably after HIV-1 infection, was associated with increased cell activation, and resulted in improved abilities to stimulate HIV-1-specific CD8 T cells. As such, our data demonstrate that type I IFN responses to HIV-1 in cDCs may play an important role in the network of immune defense mechanisms in elite controllers, and delineate specific connections between innate HIV-1 immune recognition and the generation of adaptive immune responses in this specific patient population.

Dendritic cells represent a relatively hostile environment for HIV-1 and do not effectively support HIV-1 replication steps, likely due to specific myeloid host proteins that effectively block HIV-1 replication steps. SAMHD1 is one of the most prominent myeloid restriction factors, and can effectively inhibit HIV-1 reverse transcription by hydrolyzing dNTPs, which are typically only available in limited amounts in resting myeloid cells. Despite evidence that SAMHD1 sequences have been under positive selection pressure [51], it is interesting that HIV-1 has not developed effective measures to counteract the antiviral activity of this host factor, suggesting that this protein may not significantly impair the ability of HIV-1 to establish a long-lasting, progressive infection of the host. Our study demonstrates the paradoxical finding that cDCs from EC are more permissive to early steps of HIV-1 replication while cDCs from patients with progressive infection are more resistant to HIV-1 infection. This corresponded to our observation that upregulation of SAMHD1 in response to HIV-1 was lower in EC, leading to more efficient reverse transcription in cDCs from these patients. Whether this reduced induction of SAMHD1 in cDC from EC represents a constitutive, cell-intrinsic aspect of cDC from these patients, or occurs as a result of specific interactions with other cell types [52] is uncertain at present and requires further investigation. Overall, these data suggest that the SAMHD1-dependent restriction of HIV-1 replication in cDCs benefits the virus more than the host, and that a higher susceptibility of cDCs to HIV-1 paradoxically allows for more effective systemic immune defense against HIV-1. Notably, ISG expression and levels of IFNα/β expression prior to *ex-vivo* infection with HIV-1 tended to be elevated in cDC from CP, consistent with higher levels of immune activation in this cohort; it is possible that this increased baseline immune activation may make cDC from these patients refractory to HIV-1-induced cell-intrinsic immune responses.

Cell-intrinsic immune responses in cDCs from EC in our study appeared to be facilitated by an accumulation of reverse transcripts. These viral products seem to represent the main viral substrate that is responsible for the induction of cellular type I IFN responses, as pharmacological inhibition of viral reverse transcripts almost completely inhibited cell-intrinsic IFN-I secretion, while inhibition of HIV-1 integration did not. These observations support previous data suggesting that single-stranded HIV-1 DNA transcripts represent the predominant HIV-1 replication product that cell-intrinsic microbial sensors recognize [27,53]. The exact reasons responsible for the accumulation of reverse transcripts in EC are not entirely clear; however, relative to the levels of reverse transcripts, HIV-1 integration in cDCs from EC was disproportionately reduced, and the ratio of reverse transcripts to integrated HIV-1 DNA was exceedingly higher in cDCs from EC compared to all other patient cohorts. Together these data suggest that cDCs from EC preferentially support a form of abortive HIV-1 infection that progresses effectively through reverse transcription, but is restricted at the level of viral integration, possibly through as of yet undefined host factors. Notably, prior studies found evidence for restriction of HIV-1 integration in resting CD4^+^ T cells from EC [54], although the underlying reason remained uncertain. The weak induction of host factors required for HIV-1 integration in cDCs from EC, such as LEDGF/p75, may represent one important aspect contributing to the accumulation of RT products in cDCs from EC, but a closer analysis of molecular mechanisms regulating and possibly restricting HIV-1 integration in cDCs from EC will likely be highly informative. In addition, it is interesting that higher levels of integrated HIV-1 DNA in CP and HAART-treated patients did not translate in higher levels of HIV-1 mRNA transcription, as determined by GFP expression; this suggests that additional restriction at the level of viral gene transcription may be operational in these patients.

An important aspect of this work is the identification of cGAS as a critical factor for the generation of cell-intrinsic type I IFN responses in primary cDCs. cGAS can act as a cytosolic microbial DNA sensor that induces interferon secretion by production of the second messenger cGAMP [43,55,56] and can play an important role for innate recognition of HIV-1 DNA in myeloid cells [42]. However, in prior studies, cGAS-dependent immune recognition of HIV-1 was mostly analyzed in monocyte-derived DCs, which are highly resistant to HIV-1 unless co-transfected with viral inhibitors of SAMHD1 [27]. Our experiments in more physiologic primary cDCs from EC demonstrated that HIV-1 infection led to a rapid induction of cGAS expression, and cGAS silencing in primary cDCs effectively inhibited type I IFN secretion. As such, our work strongly suggests that cGAS can sense HIV-1 DNA under physiologically relevant conditions, and that a more rapid induction of cGAS expression facilitates the generation of cell-intrinsic type I IFN secretion in cDCs from EC. Notably, STING, an adaptor molecule that acts downstream of cGAS-dependent immune recognition [43,57], also tended to be more effectively induced in cDCs from EC, suggesting combined activity of the cGAS/STING pathway for HIV-1 immune recognition in these patients. Interestingly, IFI16, which can act as a cellular sensor for HIV-1 DNA in lymphoid CD4^+^ T cells and induces pyroptosis after abortive infection with HIV-1 [45], was similarly induced in cDCs from all cohorts early after infection, and therefore unlikely to be responsible for the early type I IFN responses observed in EC.

While the role of highly-functional HIV-1-specific CD8 T cells for HIV-1 immune defense in elite controllers is supported by a large number of studies [58–61], the ontogeny of these effective immune responses still remains largely obscure. Our data suggest that more efficient cell-intrinsic responses against HIV-1 in cDCs from EC translate into an enhanced ability to stimulate HIV-1-specific T cell responses. This may correspond to prior description to specific expression patterns of immunoregulatory receptors on cDC from EC [62]. As such, more effective cell-intrinsic IFN secretion in cDCs may represent a distinguishing feature of elite controllers, and a key mechanism supporting the evolution of effective adaptive cellular immune responses. Yet, growing evidence suggests that the ability of elite controllers to maintain undetectable levels of viral replication is associated with increased levels of immune activation [63,64], which may put patients at risk for higher frequencies of cardiovascular events and accelerated immune aging. Therefore, it is tempting to speculate that improved abilities for cytosolic microbial immune recognition may represent a constitutive characteristic of ECs that predisposes them for more potent immune activity against HIV-1, at the expense of elevated generalized levels of immune activation. An improved understanding of interactions between innate immune recognition in cDCs, immune activation and the evolution of adaptive CD8 T cell responses in elite controllers will therefore be necessary for designing clinical strategies aiming at inducing an elite controller-like phenotype in broader populations of HIV-1 patients.

## Materials and Methods

### Study participants

HIV-1 elite controllers who had maintained undetectable levels of HIV-1 replication for a median of 5 years (range 2–14) in the absence of antiretroviral therapy (EC, n = 26, median VL: <49 copies/ml, median CD4 T cell counts: 835 cells/μl, range 444–2459 cells/μl), untreated chronic progressors (CP, n = 26, median VL: 9010 copies/ml, range 1360–111000; median CD4 T cell counts: 471 cells/μl, range 149–1098 cells/μl), HAART-treated chronically HIV-1-infected patients with suppressed HIV-1 viremia (HAART, n = 23, median VL: <49 copies/ml; median CD4 T cell counts: 710 cells/μl, range 109–1325 cells/μl) and HIV-1 seronegative healthy persons (Neg, n = 26), were recruited for this study.

### Ethics statement

All subjects gave written informed consent and the study was approved by the Institutional Review Board of Massachusetts General Hospital/Partners Healthcare.

### Viruses and constructs

GFP-encoding R5-tropic Ba-L HIV-1 viruses and GFP-encoding Δenv NL4-3 HIV-1 viruses pseudotyped with vesicular stomatitis virus G envelope protein (VSV-G) [27,65,66], were kindly provided by Dr. Dan Littman (New York University, New York, New York, USA). Viral particles were produced by transfecting 293T cells with the respective HIV-1 plasmids and, if applicable, with pCG-VSV-G, using TransIT-293 (Mirus) in OptiMEM per the manufacturer’s instructions. For control purposes, 293T cells were also transfected with Salmon Sperm DNA solution (Invitrogen). Supernatants were harvested 48 hours after transfection, centrifuged, filtered and treated with DNase I (20 U/ml) at room temperature for 1 hour, and stored at −80°C.

### Isolation and purification of DC

BDCA1^+^ cDCs were purified from total PBMC suspensions by MACS using anti-human BDCA-1 Kit and MS and LD columns (Miltenyi Biotec) (purity > 90%) for subsequent analysis.

### Ex-vivo infection assays and cell culture

PBMC or isolated cDC, without prior *in vitro* culture with activating agents, were infected with GFP-encoding VSV-G peudotyped HIV-1 virus (MOI = 2.4) or R5-tropic HIV-1 (Ba-L, MOI = 0.4) for 2 or 4 hours, respectively, at 37°C in the presence of 5μg/ml Polybrene (Sigma). Cells cultured with media only or with 2μg/ml TLR3 ligand Poly (I:C) were used as negative or positive control, respectively. Where indicated, PBMCs were cultured in the presence of 30ng/ml IFNβ (PeproTech). After two washes, cells were plated at 5×10^5^ cells per well in a 24-well plate. Where indicated, the infection assays were performed in the presence of 50nM AZT or 100nM Efavirenz or 30μM Raltegavir to specifically block generation of either early or late HIV-1 RT-products or integration, respectively. All antiretroviral agents were obtained from the NIH AIDS Reagent Program (https://www.aidsreagent.org/program_info.cfm). In addition, monocyte derived dendritic cells were generated as previously described [62,67] and analyzed similarly after infection with HIV-1 for control purposes.

### Gene expression analysis

cDNA was synthesized from total RNA obtained from purified BDCA1^+^ cDCs using the *mir*Vana Isolation Kit (Life Technologies). Subsequently, expression of selected gene transcripts (IFNα/β, SAMHD1, TREX1, TPNO3 and LEDGF/p75) was analyzed by semiquantitative PCR using the Taqman gene expression assay (Life Technologies) with standardized primers/probes, and normalized to the expression of the housekeeping gene *ACTB* (encoding β-actin). In addition, expression of 28 interferon-stimulated genes (ISG) and 3 intracellular DNA sensors (cGAS, STING, IFI16) was analyzed using customized 96-well plate TaqMan gene expression assays (Life Technologies) using a ViiA 7 instrument (Life Technologies).

### Flow cytometry

At 24, 48 and 96 hours post-infection, PBMC were stained with LIVE/DEAD cell blue viability dye (Invitrogen, Carlsbad, CA) and monoclonal antibodies directed against CD11c (Biolegend), CD14 (BD), CD40, CD83, CD86, HLA-DR (Biolegend), and BDCA-1 (Miltenyi Biotec) and subsequently analyzed on a Fortessa cytometer (BD Biosciences, San Jose, CA). For intracellular cytokine staining, cells were treated with a commercial fixation/permeabilization kit (BioLegend) according to the manufacturer’s protocol. Data were analyzed with FlowJo software (Tree Star). cDCs were identified from bulk PBMCs as a population of viable CD14^-^ lymphocytes expressing high levels of CD11c and HLA-DR and the cDC specific marker BDCA-1.

### Analysis of HIV-1 replication products

Early and late HIV-1 reverse transcripts and 2-LTR circles were amplified from cell lysates as previously described [68]. Integrated HIV-1 DNA was determined using nested PCR with Alu-LTR primers as previously described [54,65,69]. Copy numbers of reverse transcripts and integrated HIV-1 DNA were obtained after extrapolation to specific standard curves generated from HIV-1-infected 293T cells (kindly provided by Dr. Bushman, University of Pennsylvania). qPCR data were normalized to relative CCR5 gene copy number. To determine the amounts of *de novo* integrated HIV-1 DNA and 2-LTR circles in our *ex vivo* infection assays, raw qPCR data were corrected to the basal levels present in cDCs from each corresponding subject. Determination of 2-LTR circle copy numbers were calculated by extrapolating qPCR values to a standard curve generated using a plasmid harboring the sequence of 2-LTR junction and the CCR5 gene (kindly provided by Dr. Mario Stevenson, University of Miami, FL) [54].

### Western blot

Isolated cDCs from indicated study cohorts were lysed in RIPA buffer (Thermo Scientific) supplemented with Halt Protease and Phosphatase inhibitors (Thermo Scientific). Lysates were normalized for protein concentration using a Bradford assay (Bio-Rad), separated by SDS-PAGE in 4–12% Tris-Glycine gels (Novex, Life Technologies) and transferred to a PVDF membrane using the iBlot Gel Transfer system (Novex, Life Technologies). Membranes were then blocked for 30min at room temperature using Odyssey Blocking buffer (LI-COR Biosciences) and incubated with 1:1000 dilutions of anti-mouse β-Actin (Abcam) and either mouse anti-SAMHD1 (Clone 1F6, Origene) or rabbit anti-cGAS antibodies (clone MB21D1, Sigma), followed by secondary hybridization with goat anti-mouse (clone IRDye 800cW) and goat anti-rabbit (clone IRDye 680RD) antibodies (Odyssey). The blots were visualized using an Odyssey imaging system (LI-COR) and the bands were quantified and normalized to β-Actin levels using Image Studio 3.1 software (LI-COR).

### siRNA-mediated gene knockdown

Knockdown of the DNA sensor cGAS and the restriction factor SAMHD1 were performed by nucleofection of primary cDC (program FF137, Amaxa 4D-Nucleofector, Lonza) with specific or scramble siRNAs (Thermo scientific) according to the manufacturer’s instructions. Nucleofected cDC were infected with VSV-G-pseudotyped HIV-1 after 16 hours, type I IFN responses and HIV-1 reverse transcripts were subsequently analyzed at 24 hours p.i. by qPCR. Efficiency of siRNA-mediated knockdown was confirmed at the mRNA and protein levels by qPCR and Western blot, respectively (Fig 4 and S5 Fig).

### Mixed leukocyte reaction assays

PBMC were cultured in the presence of media or VSV-G-pseudotyped HIV-1 in the absence or presence of described concentrations of AZT or EFV or Raltegavir for 24 hours. Afterwards, BDCA1^+^ cDCs were purified by immunomagnetic enrichment and mixed with allogeneic total peripheral blood T lymphocytes previously stained with 5μM carboxyfluorescein succinimidyl ester (CFSE; Invitrogen) at a T:DC ratio of 4:1. As a control, T cells were also cultured in the presence of media only or 2.5μg/ml PHA and 50IU/ml IL-2. After incubation for 6 days, cells were washed, stained with viability dye and anti-CD4 and anti-CD8 antibodies (Biolegend, San Diego, CA), and CFSE dilution on CD4 and CD8 T cell subpopulations was analyzed by flow cytometry using a Fortessa flow cytometer.

### *In vitro* stimulation of HIV-1 specific T cell responses

PBMC from HLA*02:01^+^ individuals were cultured in the presence of media or VSV-G-HIV-1 for 48 hours. Subsequently, BDCA1^+^ cDCs were isolated as previously described and co-cultured with a CD8^+^ T cell clone specific for the HLA*02:01 restricted gag peptide SL9 (SLYNTVATL) at a T:DC ratio of 4:1. After 1 hour of initial incubation, cells were cultured for additional 16 hours in the presence of Brefeldin A (BioLegend) and Monensin (Golgi Stop; BD Biosciences). Subsequently, intracellular expression of IFNγ (BioLegend, San Diego, CA) in the CD8 T cell clone was analyzed by flow cytometry after intracellular cytokine staining.

### Statistical analysis

Significance of phenotypic differences between the different patient cohorts were assessed using Mann Whitney U tests or Wilcoxon matched-pairs signed-rank test. When appropriate, statistical analysis was corrected for multiple comparisons using a Kruskal-Wallis test with post-hoc Dunn’s test or the Bonferroni correction. To investigate expression patterns of ISGs, we used unsupervised complete linkage hierarchical cluster analysis, which groups samples together by the expression similarity, and gene coexpression network analysis, which identifies the co-expression relationships among genes from different patient cohorts, by Pearson’s correlation coefficients [70].

## Supporting Information

S1 Fig Expression of costimulatory molecules in cDCs from EC after *ex-vivo* infection with HIV-1. (A): Representative flow cytometry histograms reflecting CD86 (upper panel), CD83 (middle panel) and CD40 surface expression (lower panel) of cDCs from HIV-negative persons (Neg), individuals with chronic progressive HIV-1 infection (CP), Elite controllers (EC) and HAART-treated HIV-1 patients (HAART) 24h after exposure to media (full grey histograms), HIV-1 (blue histograms) or Poly I:C (purple histograms). Black histograms represent background levels for each marker defined by FMO controls. (B): Mean Fluorescence Intensity (MFI) values of surface expression of CD86, CD83 and CD40 on cDCs from the different study cohorts 24h (upper panels) or 48h (lower panels) after exposure to media only (Med), HIV-1 (HIV) or to the TLR3 ligand poly(I:C) (PIC). Data on plots represent raw MFI values. Differences within and between study cohorts were tested for statistical significance using Wilcoxon matched-pairs signed rank test and Mann Whitney test, respectively. Horizontal lines represent the median for each specific cohort and experimental condition. Bonferroni correction was used for multiple comparisons; * p<0.05; ** p<0.01. (C): MFI values of surface expression of CD86, CD83 and CD40 on HIV negative cDCs after 24h in culture of media (Med) or the indicated concentrations of IFNβ. The plots correspond to a single experiment. (D): MFI values of surface expression of CD86, CD83 and CD40 on cDCs from the different study cohorts after 24h of culture in media only (Med) or supplemented with 30ng/ml of IFNβ. Plots represent the summary of n = 3 independent experiments. Horizontal lines represent mean values.
(EPS)

S2 Fig Susceptibility of MDDCs and primary cDCs to *ex vivo* infection with VSV-G-pseudotyped or R5-tropic HIV-1. (A): Flow cytometry plots showing proportions of GFP^+^ MDDC at 24, 48 and 96 hours after exposure to GFP-encoding VSV-G-pseudotyped HIV-1. Numbers in plots indicate the percentage of GFP^+^ cells. One representative experiment out of four is shown. (B–C): Proportions (B) and GFP MFI (C) of GFP^+^ primary cDCs from indicated study cohorts 96 hours after exposure to R5-tropic HIV-1 virus. Horizontal lines represent the median for each specific cohort and experimental condition. Differences were tested for statistical significance using a Kruskal Wallis test with post-hoc Dunn’s test (** p<0.01; *** p<0.001) or using Mann Whitney U test (# p<0.05; ## p<0.01). (D): Proportions of cDCs contained in CD14^-^ lymphocytes from Neg, CP, EC and HAART after 96h of infection with a VSV-G-pseudotyped GFP-enconding HIV-1 virus.
(EPS)

S3 Fig Rapid accumulation of HIV-1 RT products in cDC from EC. Detection of early and late HIV-1 reverse transcripts (RT) in cDCs from healthy individuals (Neg, blue), HIV-1^+^ chronically infected persons (CP, orange), elite controllers (EC, green) and patients undergoing anti-retroviral therapy (HAART, purple) at 24 hours after *ex vivo* infection with HIV-1. Horizontal lines represent the median for each specific cohort and experimental condition. Differences were tested for statistical significance using a Kruskal Wallis test with post-hoc Dunn’s test (* p<0.05; ** p<0.01) or using Mann Whitney U test (# p<0.05).
(EPS)

S4 Fig SAMHD1 protein levels in primary cDCs from different study cohorts. Western blot analysis of SAMHD1 (upper panel) and β-Actin (lower panel) protein levels in isolated BDCA1^+^ cDCs from HIV-negative persons (Neg), individuals with chronic progressive HIV-1 infection (CP), Elite controllers (EC) 48h after exposure to medium (Med), HIV-1 (HIV-1) or Poly I:C (P.I:C). (A) shows results from representative patients from different experiments, (B) summarizes cumulative data from n = 5 study subjects from each cohort.
(EPS)

S5 Fig cGAS is required to induce type I IFN responses in primary cDCs. (A): Spearman correlations between induction of IFNβ expression and induction of cGAS (left) and STING (right) expression levels in cDCs 48 hours after exposure to HIV-1. (B): Flow cytometry analysis of viability on primary cDCs 24h after nucleofection with scramble- (SC) or cGAS-specific (cGAS) siRNA. Numbers of dot plots represent the percentage of viable CD11c-positive viability dye-negative DCs. (C): Efficacy of siRNA-mediated knockdown of cGAS expression in primary cDC. Data indicate mRNA (left panel) and protein (right panel) expression levels of cGAS in cDCs nucleofected with scramble- (SC) or cGAS-specific siRNAs. Relative inhibition of cGAS mRNA expression after knockdown is indicated. (* p<0.05, Wilcoxon matched-pairs signed rank test). (D): IFNβ mRNA levels present on cDCs nucleofected with SC- or cGAS-specific siRNAs and cultured in the presence of media (Med) or Poly I:C (PIC). Horizontal lines represent the median for each specific cohort and experimental condition.
(EPS)

S1 Table Fold change in expression of 28 ISGs in cDCs after HIV-1 infection. (DOCX)

S2 Table Basal levels of 28 ISGs in cDCs from EC and CP. (DOC)
