# Supplementary material for: Potent Cell-Intrinsic Immune Responses in Dendritic Cells Facilitate HIV-1-Specific T Cell Immunity in HIV-1 Elite Controllers
Source: PLoS Pathog. 2015 Jun 11;11(6):e1004930. doi: 10.1371/journal.ppat.1004930 (PMC4466270; doi:10.1371/journal.ppat.1004930)
Supplement: S1 Table — (DOCX) [file ppat.1004930.s006.docx]

**S1_Table. Fold change in expression of 28 ISGs in cDCs after HIV-1 infection.**

| **Gene ID** | **Median Rq HIVneg DC** | **P value** | **Median Rq**  **CP DC** | **P value** | **Median Rq**  **EC DC** | **P value** |
| --- | --- | --- | --- | --- | --- | --- |
| **ADAR*** | **1.25** | *0.31* | **1.37** | *0.09* | **3.09** | ***0.03*** |
| **AIM2*** | **0.67** | *1.00* | **1.55** | *0.22* | **6.89** | ***0.03*** |
| **IFI6** | **1.23** | *0.06* | **3.76** | *0.06* | **3.10** | *0.09* |
| **IFI27** | **1.36** | *0.43* | **3.21** | ***0.03*** | **18.19** | *0.06* |
| **IFI30** | **0.91** | *0.56* | **0.73** | *0.22* | **0.93** | *1.00* |
| **IFI35** | **1.50** | ***0.03*** | **2.39** | *0.06* | **5.08** | *0.09* |
| **IFI44** | **1.14** | *0.68* | **3.30** | *0.06* | **22.31** | *0.06* |
| **IFI44L** | **5.97** | *0.22* | **4.53** | *0.06* | **11.78** | *0.06* |
| **IFIT1** | **4.42** | *0.09* | **2.05** | *0.15* | **4.28** | *0.15* |
| **IFIT2** | **1.62** | *0.22* | **2.75** | *0.16* | **2.96** | *0.31* |
| **IFIT3** | **2.78** | ***0.03*** | **5.44** | *0.06* | **5.18** | *0.09* |
| **IFITM1** | **1.03** | *0.68* | **4.50** | *0.16* | **6.71** | *0.09* |
| **IFITM2** | **1.01** | *0.68* | **1.35** | *0.31* | **1.36** | *0.43* |
| **IFITM3** | **1.77** | *0.16* | **2.68** | *0.06* | **2.76** | *0.09* |
| **IFIH1** | **2.40** | ***0.03*** | **2.60** | *0.06* | **3.71** | *0.06* |
| **IRF1** | **1.59** | *0.31* | **1.92** | *0.15* | **4.13** | *0.06* |
| **IRF2*** | **1.59** | *0.31* | **1.29** | *0.31* | **3.14** | ***0.03*** |
| **IRF3** | **1.00** | *1.00* | **1.39** | *0.43* | **2.01** | *0.15* |
| **IRF4** | **1.03** | *0.56* | **1.72** | ***0.03*** | **1.88** | *0.09* |
| **IRF7** | **1.92** | ***0.03*** | **2.35** | *0.09* | **5.47** | *0.09* |
| **ISG15** | **4.86** | ***0.03*** | **4.09** | *0.06* | **9.36** | *0.06* |
| **MX1** | **1.94** | ***0.03*** | **2.47** | *0.06* | **8.97** | *0.06* |
| **MX2** | **1.57** | *0.16* | **2.69** | *0.11* | **4.30** | *0.16* |
| **OAS1** | **2.63** | ***0.03*** | **3.68** | *0.06* | **2.63** | *0.06* |
| **PSME1*** | **0.94** | *0.56* | **0.99** | *1.00* | **1.83** | ***0.03*** |
| **SP110** | **1.28** | *0.56* | **1.67** | *0.06* | **2.77** | *0.16* |
| **SUN2** | **1.24** | *0.56* | **0.80** | *0.40* | **1.71** | *0.09* |
| **TRIM5** | **1.10** | *0.56* | **1.11** | *0.69* | **3.16** | *0.16* |

*** represents significant differentially expressed genes between EC and HIV negative individuals.**
