# Supplementary material for: Potent Cell-Intrinsic Immune Responses in Dendritic Cells Facilitate HIV-1-Specific T Cell Immunity in HIV-1 Elite Controllers
Source: PLoS Pathog. 2015 Jun 11;11(6):e1004930. doi: 10.1371/journal.ppat.1004930 (PMC4466270; doi:10.1371/journal.ppat.1004930)
Supplement: S2 Table — (DOC) [file ppat.1004930.s007.doc]

**S2_Table. Basal levels of 28 ISGs in cDCs from EC and CP.**

| **Gene ID** | **Median Rq HIVneg DC** | **Median Rq**  **CP DC** | **Median Rq**  **EC DC** | **P value**  **CP vs EC** | **P value**  **Neg vs CP** | **P value**  **Neg vs EC** |
| --- | --- | --- | --- | --- | --- | --- |
| **ADAR** | 0.01348 | 0.01302 | 0.005608 | *0.9372* | *0.5887* | *0.3095* |
| **AIM2** | 0.0005666 | 0.001886 | 0.0002711 | *0.5887* | *0.6991* | *0.1797* |
| **IFI6** | 0.02301 | 0.007752 | 0.008800 | *0.9372* | *0.0649* | *0.1797* |
| **IFI27*** | 0.003749 | 0.001999 | **0.0005338** | ***0.0043*** | *0.3095* | ***0.0260*** |
| **IFI30** | 0.2520 | 0.3706 | 0.09276 | *0.6991* | *1.0000* | *0.5887* |
| **IFI35** | 0.008397 | 0.007953 | 0.004161 | *1.0000* | *1.0000* | *0.3095* |
| **IFI44*** | 0.004108 | 0.002223 | **0.0005909** | *0.2403* | *0.0931* | ***0.0152*** |
| **IFI44L** | 0.001262 | 0.0007586 | 0.0008626 | *0.9372* | *0.6991* | *0.9372* |
| **IFIT1** | 0.0005277 | 0.0002789 | 0.0003273 | *0.4848* | *0.4848* | *0.5887* |
| **IFIT2** | 0.0005207 | 0.0004438 | 0.0003549 | *0.8182* | *0.8182* | *0.5887* |
| **IFIT3** | 0.001913 | 0.0006208 | 0.001251 | *0.3095* | *0.0931* | *0.5887* |
| **IFITM1** | 0.01364 | 0.005669 | 0.005202 | *0.9372* | *0.2403* | *0.4848* |
| **IFITM2** | 0.001950 | 0.001943 | 0.001228 | *0.3095* | *0.5887* | *0.1797* |
| **IFITM3** | 0.01611 | 0.01262 | 0.005902 | *0.4848* | *0.2403* | *0.2403* |
| **IFIH1** | 0.001474 | 0.0009955 | 0.0008781 | *0.9372* | *0.3939* | *0.2403* |
| **IRF1** | 0.06452 | 0.1018 | 0.02202 | *0.6991* | *0.8182* | *0.8182* |
| **IRF2** | 0.01547 | 0.01328 | 0.004326 | *0.9372* | *0.5887* | *0.4848* |
| **IRF3** | 0.003290 | 0.002770 | 0.001203 | *0.8182* | *0.5887* | *0.3095* |
| **IRF4** | 0.005548 | 0.003345 | 0.004921 | *0.5887* | *0.2403* | *0.5887* |
| **IRF7** | 0.01437 | 0.01099 | 0.005407 | *0.5887* | *0.9372* | *0.2403* |
| **ISG15** | 0.005975 | 0.003302 | 0.003008 | *0.5887* | *0.6991* | *0.4848* |
| **MX1** | 0.02414 | 0.02023 | 0.01241 | *0.6991* | *0.5887* | *0.1797* |
| **MX2** | 0.006157 | 0.004407 | 0.004062 | *0.7308* | *0.3290* | *0.6389* |
| **OAS1** | 0.006757 | 0.003161 | 0.002658 | *1.0000* | *0.0649* | *0.2403* |
| **PSME1** | 0.01260 | 0.008286 | 0.003253 | *0.6991* | *0.4848* | *0.2403* |
| **SP110** | 0.001128 | 0.0009355 | 0.0009318 | *0.8357* | *0.6623* | *1.0000* |
| **SUN2** | 0.02224 | 0.02945 | 0.005709 | *0.8182* | *0.9372* | *0.4848* |
| **TRIM5** | 0.001816 | 0.002380 | 0.0007439 | *0.9372* | *0.5887* | *0.1797* |

*** represents significant differentially expressed genes between EC and HIV negative or CP individuals.**
